# Signaling pathways in the regulation of cancer stem cells and associated targeted therapy

**DOI:** 10.1002/mco2.176

**Published:** 2022-10-05

**Authors:** Wang Manni, Wu Min

**Affiliations:** ^1^ Department of Biotherapy, Cancer Center, West China Hospital Sichuan University Chengdu P. R. China; ^2^ Department of Biomedical Sciences, School of Medicine and Health Sciences University of North Dakota Grand Forks North Dakota USA

**Keywords:** cancer stem cells, CAR‐T therapies, inhibitors, signal pathway

## Abstract

Cancer stem cells (CSCs) are defined as a subpopulation of malignant tumor cells with selective capacities for tumor initiation, self‐renewal, metastasis, and unlimited growth into bulks, which are believed as a major cause of progressive tumor phenotypes, including recurrence, metastasis, and treatment failure. A number of signaling pathways are involved in the maintenance of stem cell properties and survival of CSCs, including well‐established intrinsic pathways, such as the Notch, Wnt, and Hedgehog signaling, and extrinsic pathways, such as the vascular microenvironment and tumor‐associated immune cells. There is also intricate crosstalk between these signal cascades and other oncogenic pathways. Thus, targeting pathway molecules that regulate CSCs provides a new option for the treatment of therapy‐resistant or ‐refractory tumors. These treatments include small molecule inhibitors, monoclonal antibodies that target key signaling in CSCs, as well as CSC‐directed immunotherapies that harness the immune systems to target CSCs. This review aims to provide an overview of the regulating networks and their immune interactions involved in CSC development. We also address the update on the development of CSC‐directed therapeutics, with a special focus on those with application approval or under clinical evaluation.

## Graphical abstract

The major signaling pathways that regulate CSCs. Amongst an array of signaling pathways aberrantly activated in cancer Wnt, JAK/STAT, PI3k/Akt/mTOR, Notch, NF‐κB, Hedgehog, and TGF‐β/Smad pathways are crucial for the of self‐renewal, cell growth, metastasis of CSCs, and angiogenesis.

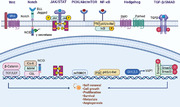

## 1 INTRODUCTION

The concept of stem cells dates back to the 18th century when scientists tried to elucidate how lower organisms developed tissues and organs.^1^ These stem cells produce daughter cells that later undergo different biological processes, either continuous self‐renewal division, or differentiation into specialized cells with a limited lifespan. Normal tissue stem cells provide a life‐long source of cells for self‐renewal of tissues, which leads us to speculate that whether stem cells are capable of deriving a malignant cell population, and this lies the foundation of cancer stem cells (CSCs) theory. CSCs are defined as a subpopulation of malignant tumor cells with selective capacities for tumor initiation, self‐renewal, metastasis, and unlimited growth into bulks.^2^

Despite decades of research on cancer treatment, it has been proved extremely challenging to achieve complete remission (CR) in cancer patients. Tumor relapse may be explained by the fact that antitumor therapeutics mainly target proliferative cancer cells but remain ineffective in quiescent CSCs. The role of CSC in tumor initiation was first identified in acute myeloid leukemia (AML). Since its isolation from a number of solid tumors and hematological malignancies, the CSC is believed to form the clonogenic core of these tumors.^3^ Growing evidence now suggests that CSCs are responsible for multiple progressive tumor phenotypes, including recurrence, metastasis, and treatment failure.^4^, ^5^ The intrinsic treatment resistance of tumors has partially attributed to the presence of the CSC subpopulation,^6^, ^7^ and may also be induced by extrinsic factors, such as treatments and environments.^8^, ^9^

Major signaling pathways are involved in the maintenance of stem cell properties and survival of CSCs, such as the Notch, Wnt, and Hedgehog (HH) pathways.^10^ There is also intricate interplay network between these signal cascades and other oncogenic pathways.^11^, ^12^, ^13^ Thus, targeting pathway molecules that regulate CSCs provides a new option for the treatment of therapy‐resistant or ‐refractory tumors. This review aims to provide an overview of the regulating networks and their immune interactions involved in CSC development. We also summarized the update on the development of CSC‐directed therapeutics, with a special focus on those with application approval or under clinical evaluation.

## 2 CHARACTERISTICS AND IDENTIFICATION MARKERS OF CSCs

### 2.1 Characteristics of CSCs

Over the past decades, a wide breadth of literature investigated the biological characteristics of CSCs, with the hope to develop CSC‐targeted strategies that eradicate treatment‐insensitive or ‐refractory tumor cells. The potent self‐renewal ability is probably the best‐characterized property of CSCs and a direct cause of tumor initiation.^14^ CSCs divide into daughter cells in a symmetrical splitting manner and ultimately lead to excessive tumor growth.^15^ Experimental data revealed the tumorigenic function of CSCs by forming new tumors with CSCs isolated from primary tumor tissues in immunodeficient mice.^16^

Another characteristic of CSCs is their differentiation ability. For instance, leukemia stem cells (LSCs), characterized by CD34‐positive expression and deficient CD38 expression, were able to differentiate into multiple cell types in SCID mice.^17^ In addition, CSCs isolated from human brains share similar surface markers CD133 and Nestin, with normal neuronal stem cells, and are thus believed to have differentiation capabilities.^18^ In normal tissues, the balance between self‐renewal and differentiation of stem cells controls the cell fate, the aberrant regulation of which lead to tumorigenesis.^19^ Interestingly, the trans‐differentiation of CSCs into other multilineage cells may also contribute to tumor formation.^20^ One such example is the trans‐differentiation of CSCs into vascular endothelial cells, leading to oncogenesis and tumor angiogenesis of glioblastoma,^21^ renal,^22^ and liver cancer.^23^

Tumor heterogeneity is one of the key reasons for therapeutic resistance.^24^ The theory of tumor heterogeneity concept dates back to the 1970s when tumors are believed to consist of multiple distinct tumor cell subpopulations.^25^ CSCs are believed to contribute to tumor heterogeneity. This cell subpopulation gives rise to cancer cells with diverse differentiation levels, which then go through sporadic mutations and environmental changes for clone selection.^26^ The aberrant differentiation programs of CSCs resemble the hierarchical compositions of normal stem cells, which ultimately lead to a hierarchical set of tumor cells.

CSCs have also been identified with surviving and expanding capacity after cytotoxic anticancer treatment, which were recently found to enrich in remaining tumor bulks following chemotherapy treatments.^27^, ^28^ For instance, after chemotherapy treatment, preleukemic DNMT3Amut hematopoietic stem cells (HSCs) are able to generate a hematopoietic hierarchy that facilitates their survival and expansion.^29^ Likewise, the transformation of differentiated tumor cells to glioma stem cells (GSCs) was often observed in temozolomide (TMZ)‐treated glioma.^30^ The underlying mechanisms for the chemoresistance caused by CSCs include epithelial‐mesenchymal transition (EMT), dormancy, and tumor environment, which we will discuss in the review.^31^, ^32^, ^33^

### 2.2 Identification markers of CSCs

The unique gene expression profile of the CSC makes it different from bulk tumor cells, which can be used as CSC‐specific identification markers. Commonly used methodologies that evaluate CSC stemness include the detection of stemness genes, surface proteins, such as CD44 and CD133, intracellular markers, such as aldehyde dehydrogenase (ALDH), and at a more macroscopic level, phenotypic assays, such as tumorsphere formation tests.^34^, ^35^, ^36^, ^37^ Table 1 shows the key markers of CSCs in solid tumors and hematological malignancies. A number of stemness genes have been reported to modulate cell stemness in embryos and adults, including the transcription factors POU class 5 homeobox 1 (POU5F1, OCT4), Nanog homeobox (NANOG), Sex‐determining region Y‐box 2 (SOX2), Kruppel‐like factor 4 (KLF4), and MYC proto‐oncogene.^38^, ^39^

**TABLE 1:** Key markers of cancer stem cells in solid tumors and hematological malignancies

CSC surface marker	Cancer types
CD4	Head and neck squamous cell carcinoma
CD9	Glioblastoma
CD10	Acute myeloid leukemia, head and neck squamous cell carcinoma
CD13	Liver, pancreatic cancer
CD15	Glioblastoma
CD19	Acute myeloid leukemia
CD20	Acute myeloid leukemia, melanoma
CD24	Breast, gastric, liver, colorectal, ovarian cancer
CD25	Chronic myeloid leukemia
CD26	Chronic myeloid leukemia
CD29 (ß1 integrin)	Breast cancer
CD33	Chronic/acute myeloid leukemia
CD34	Acute myeloid leukemia
CD36	Chronic myeloid leukemia, glioblastoma
CD44 (and its variants)	Breast, lung, gastric, liver, colorectal, prostate, bladder, esophageal, ovarian, pancreatic, cervical cancer, glioblastoma
CD47	Liver cancer
CD49f	Breast, gastric, colorectal, cervical cancer, glioblastoma
CD54	Gastric cancer
CD61	Breast cancer
CD70	Breast cancer
CD71	Acute myeloid leukemia
CD87	Lung cancer
CD90	Breast, lung, gastric, liver, esophageal, pancreatic cancer, glioblastoma
CD98	Head and neck squamous cell carcinoma
CD117	Chronic myeloid leukemia, prostate, ovarian, lung cancer
CD123	Chronic/acute myeloid leukemia
CD133	Breast, lung, gastric, liver, colorectal, prostate, ovarian, pancreatic, cervical cancer, melanoma, glioblastoma, head and neck squamous cell carcinoma
CD166	Lung, colorectal, ovarian cancer
CD206	Colorectal, liver cancer
CD271	Melanoma, head and neck squamous cell carcinoma
Ov6	Pancreatic cancer
CXCR4	Breast, gastric, prostate, renal, lung cancer
EpCAM	Breast, lung, gastric, liver, colorectal, prostate, pancreatic cancer
LGR5	Breast, gastric, colorectal cancer
ProC‐R	Breast cancer
IL1RAP	Chronic myeloid leukemia
LINGO2	Gastric cancer
CLL‐1	Acute myeloid leukemia
TIM3	Acute myeloid leukemia
L1CAM	Glioblastoma
EGFR	Glioblastoma
ABCG2	Lung, cervical cancer
CK17	Cervical cancer

As for the surface markers that are differentially expressed on CSCs,^40^, ^41^some of the markers are not unique to CSCs and may also be present on normal stem cells.^42^, ^43^ The first reported CSC surface markers were CD34 and CD38 proteins which are used to identify HSCs and LSCs in acute myelogenous leukemia.^44^ ABCG2 is a phenotypic marker for CSCs^45^ in ovarian,^46^ hepatic,^47^ breast,^48^ lung cancer,^48^ and AML.^49^ ABCG2 is believed to associate with therapeutic resistance caused by CSC and is highly expressed in side population (SP) cells (defined as the cell fraction that excludes Hoechst DNA binding dye).^50^ The fluorescence‐activated cell sorting (FACS)‐based SP sorting method is commonly used for CSCs isolation.

CD133 (Prominin 1), coded by the gene PROM1, was initially considered a surface marker for colorectal CSCs,^51^, ^52^ and was later confirmed of its expression on CSCs of multiple origins. However, as recent evidence suggested that CD133^‐^ cell subsets were capable of inducing tumorigenesis, CD133 positivity may not necessarily indicate CSC stemness.^53^ In addition, CD133^+^ cells were also found to promote tumor metastasis.^54^ Results from a single‐cell proteomic profiling analysis revealed a higher level of CD133 compared with other stemness markers, such as NANOG and ALDH1A1, in lung cancer cells with EMT phenotypes.^55^ Given that epithelial‐mesenchymal plasticity of CSCs is a potential trigger for tumor metastasis,^56^ the coexpression of CD133 and other stemness markers may be used to identify cells with stemness characteristics.

CD44, also known as P‐glycoprotein 1, is a transmembrane glycoprotein and cell surface adhesion receptor for hyaluronic acid (HA) and osteopontin (OPN).^57^ Accumulating evidence has suggested that stemness markers, including SOX2, NANOG, and OCT4, are highly expressed in CD44^+^ cell fraction.^58^ Though the downstream targets of CD44 remain incompletely defined, known cancer‐associated signaling pathways include Rho GTPases, Ras‐MAPK, and phosphatidylinositol‐3‐kinase (PI3K)/AKT cascades.^59^ The alternative splicing of the CD44 gene leads to multiple variants, and among all the variants, CD44v is expressed in epithelial cells and critical for maintaining stemness.^60^ Although HA is a ligand for all forms of CD44, OPN only interacts with CD44v rather than CD44s.^61^ The crosstalk between HA and CD44 regulates a number of biological processes leading to tumor cell stemness, invasion, and metastasis. For instance, HA binds to CD44 and triggers the NANOG‐STAT3 pathway activation, leading to the self‐renewal of ovarian cancer cells.^62^ On the other hand, OPN, enriched in gliomas, has also been found to promote tumor cell stemness via the OPN‐CD44 axis.^63^ This evidence suggests that the downstream activities of CD44 might depend on the selection of variants by the ligands. Thus, CD44 should not be simply addressed as a marker for CSCs, the functions of which rely on its preference of variants and ligands present in the microenvironment.

However, the surface markers of CSCs may vary according to tumor types and the cell of tumor origin, demonstrating high heterogeneity between tumors or even among cells within the same tumor.^35^, ^64^ Examples include breast CSCs, which frequently display different surface marker patterns, such as CD44^+^, CD24^−^, and ALDH^+^,^65^, ^66^ and melanoma stem cells that can either be CD271^−^ or CD271^+^.^67^ Such heterogeneity of CSC surface markers has also been reported in glioblastoma,^68^ prostate cancer,^69^ and lung cancer.^70^ Moreover, the expression of CSC biomarkers is not constant and may change depending on the external environment. Higher CD133 expression was observed on stem cell‐like pancreatic cancer cells under hypoxia^71^ and the enzymatic dissociation alters the retention of surface CD133 on glioma cells.^72^

To overcome the challenge caused by constantly changing surface markers for CSCs, researchers then used nonmembrane biomarkers for CSC identification, and the ALDH represents one of its kind. The expression of ALDH is often found in normal stem cells. Highly active ALDH1 can be used to identify CSCs in breast, bladder, lung cancer, embryonal rhabdomyosarcoma, and head and neck squamous cell carcinoma.^73^ ALDH1 expression is also related to the therapeutic resistance of cancer cells to chemotherapies.^74^ However, subpopulations of breast cancer CSCs appeared to be ALDH‐negative.^75^

EMT allows a polarized epithelial cell to escape from its interaction with the basement membrane and transform into mesenchymal cell phenotypes.^76^ Thus, cancer cells that undergo EMT are more prone to invasion and metastasis.^77^ A subpopulation of breast cancer CSCs showed both EMT and CSC markers CD44, ABCG2, and ALDH1A1/3, which might serve as identification markers for metastasis‐initiating cells.^78^ In addition, another CSC subpopulations, which are ITGA6‐positive but deficient in ABCG2 and ALDH1A1/3, are referred to as non‐CSC cells with metastatic abilities but no oncogenic activities.^78^ Previous studies reported a fraction of ITGA6^+^ cells exhibiting epithelial characteristics involved in metastasis.^79^ In colon cancer, ITGA6^+^ cells are included in the CD44^+^/CD133^+^ cell population, representing the metastatic non‐CSCs.^80^

## 3 SIGNALING PATHWAYS REGULATING CSCs

**FIGURE 1:**
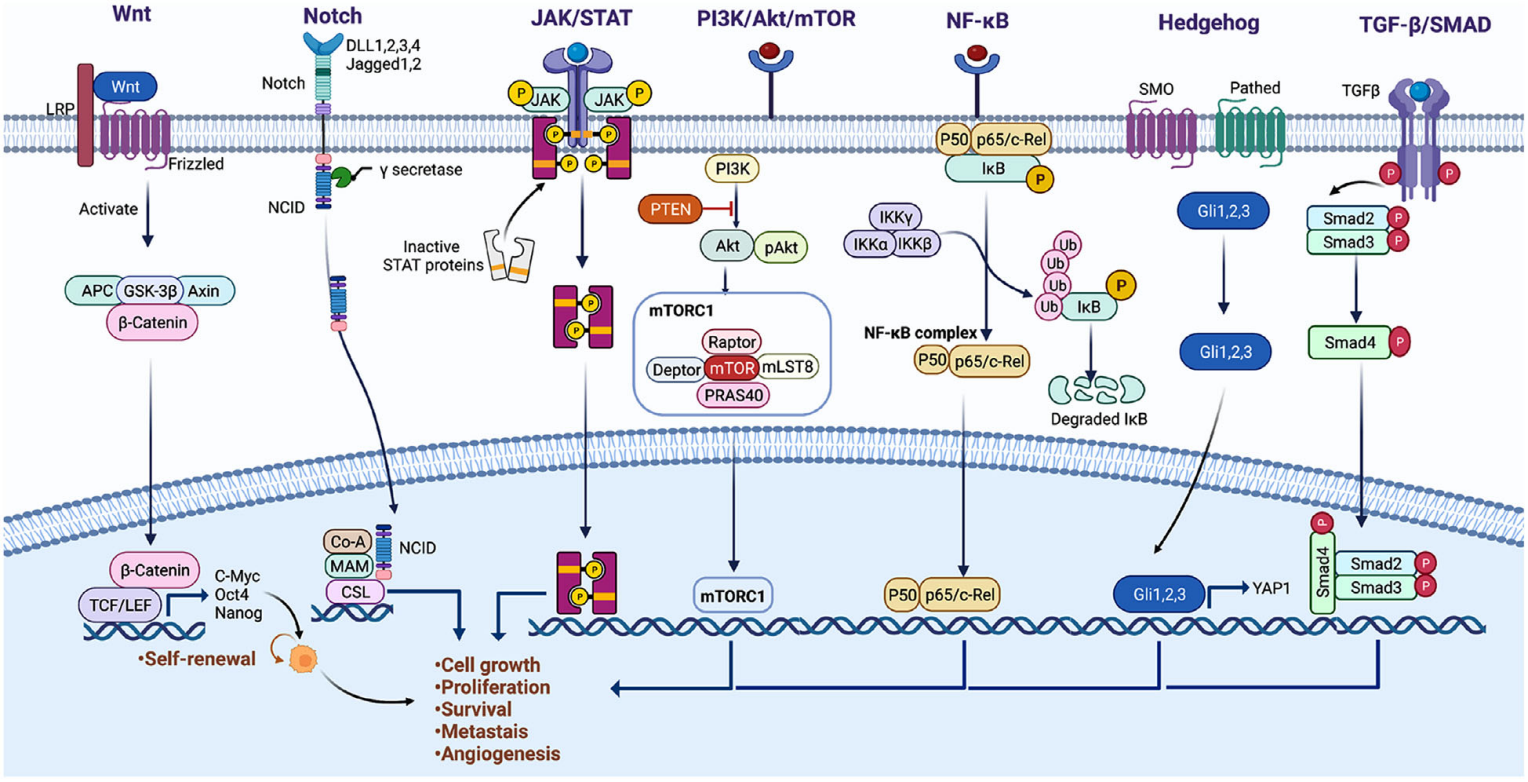
Overview of major signaling pathways that regulate CSCs. Among an array of signaling pathways aberrantly activated in cancer Wnt, JAK/STAT, PI3k/Akt/mTOR, Notch, NF‐κB, Hedgehog, and TGF‐β/Smad pathways are crucial for the self‐renewal, cell growth, metastasis of CSCs, and angiogenesis. (Figures built with biorender.com)

The homeostasis of normal stem cells is modulated by an intricate signaling network, the aberrant activation or repression of which promotes oncogenic transformation. These aberrations lead to the self‐renewal and differentiation properties of CSCs, conferring stemness to the cancers. Like their normal counterparts, CSCs also rely on these signaling pathways for survival and stemness maintenance. In this review, we classified these molecular pathways as extrinsic or intrinsic signals (Figure 1).

### 3.1 Intrinsic signaling pathway in CSCs

#### 3.1.1 Wnt signaling

The Wnt pathway is highly conserved and has long been identified as a key regulator of embryonic development and tissue homeostasis.^81^, ^82^ The abnormal activation of Wnt signaling elements, including adenomatous polyposis coli (APC), Axin, β‐catenin, and Wnt1, is frequently found in a wide range of malignancies, which is related to cancer initiation and progression.^83^, ^84^, ^85^, ^86^, ^87^ The canonical Wnt pathway is β‐catenin dependent and the noncanonical Wnt pathway does not rely on β‐catenin.

In the context of CSC regulation, the canonical Wnt signaling appears rather important in maintaining stem cell‐like traits of tumor cells.^88^ Canonical Wnt signaling is critical for maintaining lung CSC properties, potentially by regulating the expression of CSC marker OCT‐4.^89^ Other CSC markers stimulated by the Wnt/β‐catenin pathway include CD24, Prom1, CD44, and ALDH1, thereby enhancing tumor stemness.^90^ These CSC markers were also upregulated in Wnt^+^ glioblastoma cells, indicating the involvement of Wnt/β‐catenin signaling in maintaining the stemness of glioblastomas.^91^ The nuclear translocation of β‐catenin also promoted the dedifferentiation of colorectal cancer (CRC) cells, which is a novel index for stemness.^92^, ^93^ Conversely, ablation of the β‐catenin gene resulted in the loss of CSC populations and complete tumor regression of squamous cell carcinoma.^94^

In CRC, both microsatellite instable and microsatellite stable cells demonstrate activated Wnt cascades, especially at the intestinal crypts.^95^ This Wnt signal activation is mainly attributed to the functional loss of a negative regulator, APC.^95^ Another mechanism for Wnt signal activation is the function loss of RNF43 caused by gene mutations, which delays the removal of Wnt receptors in the intestinal crypt.^96^ Though 5‐fluorouracil (5‐FU), the first‐line treatment for CRC, is able to inhibit tumor growth, a recent study revealed that 5‐FU might induce CSC activation via the WNT/β‐catenin signaling pathway and thus cause chemoresistance in CRC patients.^97^ Consistent with this finding, Wnt/β‐catenin pathway activation induced by m6A modification‐Sec62‐β‐catenin promotes stemness and chemoresistance of CRC.^98^

The activated Wnt/β‐catenin pathway is enriched in more than half of breast cancers and indicates a poor prognosis.^99^ The expression of the activated β‐catenin protein is also upregulated in breast CSCs. The inhibition of β‐catenin signaling significantly prevented tumor formation and metastasis in HER2‐overexpressing breast cancer cells.^100^

Other cancer types of c regulated by WNT signaling include renal cancer, where the proliferation and self‐renewal of CSCs were significantly impaired by WNT inhibition,^101^ lung adenocarcinoma, and were activated by Wnt/β‐catenin and Notch signaling. Hypoxia‐inducible factor‐1ɑ (HIF‐1ɑ)‐regulated miR‐1275 maintains stem cell‐like phenotypes and promotes the progression of LUAD simultaneously.^102^ These results collectively suggest the essential role of β‐catenin in sustaining CSC phenotypes.

#### 3.1.2 Notch signaling

Notch signaling is a genetically conserved pathway involved in the embryonic development of the central nervous system, heart, and multiple other organs.^103^, ^104^ Notch signal is also important to the initiation and progression of cancer. In mammalian cells, four Notch transmembrane receptors (Notch 1–4) and five membrane‐bound cell surface ligands (JAG 1 and 2, DLL 1, 3, and 4) have been reported.^105^ Following the ligand‐receptor interaction, the active fragment of Notch receptors, the Notch intracellular domain (NICD), is released via proteolysis. Upon activation, the NICD‐CSL complex was formed following the nuclear translocation of NICD, which then recruits MAML and p300 coactivators, activating the downstream genes of Notch signals Hes‐1, c‐Myc, HER2, NF‐κB, cyclin‐D1, and p21.^106^

Hyperactivation of Notch signaling is significantly correlated with the maintenance of CSC characteristics in various cancer types, including breast,^107^, ^108^ colon,^109^ pancreatic,^110^ hepatic,^111^ cervical,^112^ and ovarian cancer.^113^ This theory was first established in medulloblastomas, where the tumor‐initiating ability of the CD133^+^ CSC population largely relied on Notch signal activation.^114^ Likewise, the inhibition of Notch2 activation dramatically reduced the number of CSCs, whereas the better‐differentiated cells remained unaffected.^115^

In hepatocellular carcinoma (HCC), higher expression of TACE/ADAM17 and Notch1 was found to predict a worse prognosis.^111^ Besides, inducible nitric oxide synthase enhances the TACE/ADAM17‐mediated Notch1 signaling, leading to the enrichment of CD24^+^CD133^+^ liver CSCs.^111^ In breast and pancreatic cancer, there was an increase in the expression levels of JAG1, JAG2, Notch1, Notch3, and Hes1, a downstream gene of Notch signaling.^116^, ^117^ The hypoxic microenvironment in breast cancer upregulated the expression of HIF‐2α, which then stimulated Notch signaling molecules NICD and promoted stem cell phenotypes, thereby facilitating chemoresistance of breast cancer cells.^107^ The hypoxic environment also upregulated Notch1 signaling in glioblastoma and increased its stem cell marker CD133 on the cell surface.^118^ Moreover, the heterogeneous metabolic signature of glioblastoma stem cells was also modulated by Notch signaling.^119^ Similar findings were obtained from the analyses of CSC populations in pancreatic cancer^120^ and myeloid leukemias,^121^ which displayed Notch‐mediated chemoresistance. Thus, Notch signaling plays a critical role in promoting therapy‐resistant CSC populations across malignancies. Owing to the significant impact of Notch signaling in CSCs leading to tumor initiation and therapy‐resistance, targeting Notch pathway molecules may be a promising strategy in the wide spectrum of cancers.

#### 3.1.3 JAK/STAT signaling

The JAK/STAT signaling pathway comprises various types of ligands, including interleukins, interferons, and hormones, and their corresponding receptors.^122^ Upon the binding of ligands to receptors, JAK proteins (JAK1‐3 and TYK2) are activated via phosphorylation, which then phosphorylates the cytoplasmic domain of receptors, recruiting the STAT family proteins.^123^ The dimerization and translocation of STATs lead to transcription regulation of downstream target genes.

Similar to Notch and Wnt signaling, JAK/STAT axis is also evolutionarily conserved and facilitates hematopoiesis, neurogenesis, and self‐renewal of normal embryonic stem cells. CSCs from hematological malignancies, such as AML, demonstrated aberrant activation of JAK/STAT signaling.^124^ The tumor‐formation ability of AML CSCs was potently inhibited in immunodeficient mice following JAK1/2 inhibitor treatment,^125^ reinforcing the promoting effect of JAK/STAT signaling on CSC stemness across a wide panel of cancers.^126^

The role of the JAK/STAT pathway in CSC regulation is best characterized by its tumor‐initiating effect in glioblastoma.^127^ In a glioblastoma model, the transforming growth factor (TGF)‐β‐activated JAK/STAT pathway induced the self‐renewal capacity and prevented the differentiation of glioma‐initiating cells derived from patient tumors, thereby facilitating tumor formation.^127^ Besides, inhibition of STAT3 in CSCs reduced the tumorsphere formation and increased the expression of the neuronal differentiation genes.^128^ Similar results were observed in breast CSCs, where STAT3 inhibitors decreased tumor growth and the abundance of CSCs.^129^ Recently, JAK‐STAT signaling was reported to modulate stemness and chemoresistance of myxoid liposarcoma.^130^ Likewise, JAK2/STAT3/CCND2 signals also control the radioresistance of CRC by regulating its stem cell persistence.^131^

The role of STAT3 in determining cell fate is well established.^132^ JAK proteins activate STAT3 via phosphorylation at Tyr705 residues. The downstream target genes of STAT3 nuclear translocation include cyclin D1, c‐Myc, and Bcl‐2. STAT3 holds profound importance in governing embryonic and adult stem cells in mice and humans.^133^ Apart from its modulation of self‐renewal of both embryonic and CSCs as an element of JAK signaling, STAT3 interacts with Notch ligands DLL1 (Delta‐like 1) to facilitate neocortical development in infancy.^134^ STAT3 also interacts with NF‐κB and HIF‐1α to enrich CD133^+^ cell populations.^135^ The activated form of STAT3 (p‐STAT3‐Tyr705) was enriched in ALDH^+^ and CD44^+^/CD24^+^ CSCs, the inhibition of which subsequently reduced stem cell phenotypes of this CSC population.^136^

#### 3.1.4 HH signaling

The HH signaling pathway was identified by the Nobel prize winner team in 1980.^137^ The HH pathway is critical to the development of multiple organs during embryogenesis.^138^ Interestingly, HH signaling remains inactive in all postnatal tissues except central nervous system (CNS), skin, hair, and teeth.^139^ The HH pathway is composed of three secreted ligand isoforms–Sonic hedgehog (Shh), Desert hedgehog, and Indian hedgehog, with their corresponding receptors being–Patched, Smoothened (SMO), and three Gli transcription factors (Glis1–3).^140^

The aberrant activation of HH signaling has been reported to support the proliferation and stemness maintenance of CSC in various cancer types, such as multiple myeloma, glioma, HCC, and chronic myeloid leukemia (CML).^141^, ^142^, ^143^ HH pathway activation is heterogeneous in multiple myeloma CSCs, with overexpression of the SMO gene and high Gli1 transcriptional activity.^144^ The SMO gene encodes Smoothened protein, the chemical inhibition of which reduced stemness and proliferation of multiple myeloma CSCs.^144^ In CML, the activation of HH signaling is with early disease progression.^145^ The restoration of SMO expression in SMO‐deficient CML animal models promoted tumor growth and led to a four‐fold increase in CSC proportions.^146^ Likewise, the overexpression of the HH signaling genes Gli1, SHH, and PATCHED1 was also present in glioma CSCs.^147^ HH signaling supports glioma tumor growth in animal models by inducing SMO‐expressing gliomasphere formation.^147^ A recent study showed that GLI1 inhibition reduced mammosphere formation of breast cancer cells. Interestingly, GLI1 inhibition resulted in a decrease in expression of YAP1, a Hippo pathway effector, suggesting a regulation activity of HH signaling on the Hippo pathway.^148^

The development of treatment resistance to cancer requires the functional support of CSC‐related HH signaling. GLI‐1 regulates oncogenesis and therapeutic resistance of colon rectal cancer,^149^ with significantly higher GLI‐1 expression observed in 5‐FU resistant CRC cells than in nonresistant cells.^150^, ^151^, ^152^ The expression of stem cell markers of CRC cells was significantly decreased by GLI‐1 inhibition, and the cell response to 5‐FU, Irinotecan, and Oxaliplatin was also resumed.^153^ In gastric adenocarcinoma, the forkhead box C1 (FOXC1) gene mediates the CSC phenotypes and tumor response to chemotherapy by regulating HH signaling.^154^

#### 3.1.5 TGF β/SMAD signaling

TGF‐βis a bifunctional regulator in cancer that represents a differentiation signal that potentially inhibits tumor initiation at an early stage.^155^, ^156^, ^157^ In contrast to tumor initiation, TGF‐β promotes the CSC‐like phenotypes of cancer cells by inducing EMT.^158^ TGF‐β binds to TGF‐β type I receptor kinase (ALK5) and triggers the Smad‐dependent canonical TGF‐β pathway.^159^ TGF‐β–ALK5 activates Smad2/3 via phosphorylation which then forms a complex with Smad4 and regulates gene transcription following nucleus translocation.^160^ The crosstalk between TGF‐β and the bioactive lipid mediator sphingosine‐1‐phosphate, a regulator of CSC expansion, is essential for cancer migration and the proliferation of breast CSCs.^161^, ^162^, ^163^

The TGF‐β‐SMAD signaling is implicated in the regulation of CSC‐like properties of CD44^+^ gastric cancer cells,^164^ HCC cells,^165^ and cervical cancer cells.^166^ In addition, CD44^+^ breast cancer cells, which are referred to as breast cancer CSCs, are frequently accompanied by activated TGF‐β signaling.^167^ TGF‐β induced the expression of EMT‐associated genes Snail and Twist in breast cancer CSCs.^168^ A recent study identified the role of TGF‐β‐SMAD signaling in maintaining CSCs in the bone microenvironment.^169^

TGF‐β is able to switch non‐CSCs into CSC states via activating ZEB1 and Snail.^170^, ^171^, ^172^ The response of CSCs to chemotherapy is associated with TGF‐β/Smad pathway activation, the suppression of which sensitizes CSCs to chemotherapy.^173^ According to a previous study, TGF‐β‐induced chemoresistance is a downstream reaction of the Hh signaling, suggesting the interaction of TGF‐β with Hh signaling.^174^ In addition, TGF‐β also demonstrated crosstalk with Notch signaling, with the synergistic promoting effect of TGF‐β and Notch1 on CSC proliferation.^175^

The hypoxic microenvironment is a positive regulator of TGF‐β activities in CSC stemness and chemoresistance.^176^ HIF‐1αinduces the expression and activation of TGF‐βand COX‐2, thereby promoting CSC enrichment.^177^ The positive feedback loop between Snail and TGF‐β is also regulated by hypoxia, which together promotes EMT and recruits CSCs.^178^

#### 3.1.6 NF‐κB signaling

There are five members in the NF‐κB protein family: p65 (RelA), RelB, c‐Rel, NF‐κB1 (p105/p50), and NF‐κB2 (p100/p52).^179^, ^180^ NF‐κB family proteins are present in the cytoplasm of both differentiated cells and stem cells.^181^ At the inactive state, NF‐κB are bound to inhibitory IκB proteins, which prevents its nuclear localization.^182^ Upon activation by various stimuli, such as lipopolysaccharide, the IkB kinase (IKK) complex (IKKα, IKKβ, and IKKγ) phosphorylates IκB proteins, resulting in their degradation.^183^ NF‐κB then translocates into the nucleus and activates the transcription of target genes involved in multiple biological processes.

Abnormal activation of NF‐κB signaling is implicated in the progression of various cancers. The role of the NF‐κB pathway in regulating CSCs was first identified in AML, where the primitive AML cells aberrantly expressing NF‐κB were referred to as potential leukemic stem cells.^184^ NF‐κB pathway participates in the viability and self‐renewal of AML stem‐like cells.^185^ Since then, growing evidence has shown elevated or constitutive NF‐κB activity in other cancer types. For instance, increased expression of total p65 and downregulation of IκBα expression were found in prostate CSCs.^186^ In addition, the CD44^+^ fraction of ovarian cancer cells displayed higher expression of major stemness genes and NF‐κB signal genes, including RelA, RelB, and IKKα.^187^ The loss of the APC gene represents a canonical alteration during tumorigenesis, which promotes the activation of NF‐κB signaling, allowing the expansion of Lgr5^+^ CSCs.^188^

NF‐κB activation mediates the tumorigenesis of glioma.^189^ Both adherent and spheroid glioma CSCs exhibited constitutive activation of the STAT3/NF‐κB signaling.^190^ Gliomasphere‐forming cells showed increased phosphorylation of p65 and sustained oncogenic activation of NF‐κB signaling.^191^ In Her2‐driven breast cancer models, the inactivation of NF‐κB pathways by IκBα‐SR decreased the tumorigenesis of luminal epithelial tumors.^192^ A genome‐wide expression analysis suggested that IκBαSR impaired stem cell expansion in breast cancer and CSC markers in transgenic tumors.^193^ IKKα activity is required for Her2‐induced oncogenesis, providing self‐renewal signals that maintain mammary tumor‐initiating cells.^194^ The underlying mechanism may be the phosphorylation of p27 by IKKα leading to its nuclear export in Her2 breast cancer cells.^195^ The expression of Dll1, a Notch ligand, promotes the tumor‐initiating abilities of breast cancer cells. It has been recently reported that NF‐κB activation is a downstream target of Dll1, which collectively contributes to a chemoresistant phenotype of breast cancer CSCs.^196^

#### 3.1.7 PI3K/AKT/mammalian target of rapamycin signaling

PI3K is an intracellular phosphatidylinositol kinase composed of a regulatory subunit p85 and a catalytic subunit p110.^197^, ^198^ AKT is a downstream effector of PI3K and has three isoforms: AKT1, AKT2, and AKT3.^199^ As a key member of the PI3K‐associated kinase protein family, mammalian target of rapamycin (mTOR) functions as a nutritional signal sensor and a regulating factor for cell proliferation.^200^ There are two protein complexes formed by mTOR. The mTORC1 is composed of mTOR, raptor, mLST8, and two negative regulators, PRAS40 and DEPTOR,^199^, ^201^ which controls cell growth in response to metabolism and nutrition signals.^202^, ^203^ The mTORC2 (mTOR complex 2) consists of mTOR, Rictor, mSin1, and mLST8. It is well established that mTORC2 activates Akt via phosphorylation at serine residue 473 and modulates stem‐like properties,^204^ whereas mTORC1 and its downstream cascades directly correlate with stem‐like properties.^205^

The activation of the PI3K/Akt/mTOR pathway is important to cancer cell growth and its therapeutic resistance.^206^ The PI3K/Akt/mTOR pathway can be activated through multiple mechanisms, such as the insulin‐like growth factor (IGF)/IGFR, ErbB, and fibroblast growth factor (FGF)/FGFR signaling.^206^ PTEN is known for its negative regulation on PI3K/AKT cascades. Data from several cancer models showed that PTEN depletion resulted in CSC expansion and increased tumor growth in mice.^207^, ^208^ Importantly, PI3K/AKT/mTOR is critical to maintaining the CSC population in various cancers, including nasopharyngeal carcinoma,^209^ glioma,^210^ pancreatic cancer,^211^ lung cancer,^212^ prostate,^213^ and breast cancer.^214^

In breast cancer, PI3K/Akt/mTOR pathway is required for the colony‐formation capacity of tumor cells and the maintenance of stem‐like properties.^214^ One underlying mechanism may be the HIF‐2α‐induced CD44 alteration that promotes CSC activation in triple‐negative breast cancer (TNBC) via PI3K/AKT/mTOR pathway.^215^ The transcriptional suppression of negative regulators of mTOR is intrinsic in luminal‐like breast cancer cells, leading to the development of CSC‐like properties.^216^ Likewise, the inhibition of mTOR in CRC cells suppressed cell stemness represented by decreased ALDH1 activity.^37^, ^217^ PI3K/AKT/mTOR signaling pathway also enhances the angiogenesis of CRC and recruitment of tumor‐associated macrophages (TAMs).^218^

The chemoresistance of hepatoma is also related to the Akt/mTOR signaling by promoting the expansion of hepatic tumor‐initiating cells.^219^ The inhibition of the PI3K/Akt/mTOR pathway overcame the chemoresistance of ovarian cancer by decreasing CSC marker expression.^220^ The radioresistance of prostate cancer is significantly associated with PI3K/Akt/mTOR signaling activation via maintaining CSC phenotypes.^221^ Moreover, prostate cancer CSCs present a feedback inhibition on AKT signaling through HIF1α, which impairs CSC metabolism and growth.^222^

It was previously suggested that CD133 expression was upregulated by mTOR signaling in gastrointestinal cancer.^223^ Similar results were obtained from hepatic cancer cells, where mTOR promotes the conversion of CD133^‐^ to CD133^+^ cells.^224^ Moreover, aberrant activation of the PI3K/Akt/mTOR pathway facilitates the stemness maintenance of NSCLC (non small cell lung cancer) cells by upregulating chemokine (C‐X‐C motif) receptor 4 (CXCR4) and the subsequent CXCR4‐stimulated STAT3 signaling.^225^

On the other hand, in gliomas, Akt but not mTOR regulates ATP binding cassette transporters (ABCG2) activity, which is referred to as stemness hallmark.^226^ Besides, PI3K inhibition restored the sensitivity to nilotinib of CML stem cells, whereas mTOR inhibition demonstrated no effect on CML.^227^

#### 3.1.8 Peroxisome‐proliferator‐activated receptor signaling

The peroxisome‐proliferator‐activated receptor (PPAR) pathway is activated following the binding of the G protein‐coupled receptor with its ligand, stimulating a cascade of signal transducers, such as adenylyl cyclase, cyclic adenosine monophosphate, and protein kinase A, which then induces the translocation of PPAR, a nuclear receptor protein that regulates the target gene expression.^228^, ^229^, ^230^ PPARα, PPARδ, and PPARγ are the three subtypes of PPAR with respective functions. In the context of a tumor, PPARs are involved in the modulation of cell proliferation, apoptosis, and survival of multiple cancers, including prostate cancer, breast cancer, glioblastoma, neuroblastoma, pancreatic cancer, hepatic cancer, leukemia, bladder cancer, and thyroid tumors, with either promoting or inhibitory effects on cancer development.^231^ PPARs have also been reported to regulate the EMT process and stem cell‐like properties of CSCs.^232^

CPT1A (Carnitine palmitoyl transferase I) and CPT2 (Carnitine palmitoyl transferase II) are two known target genes of PPARα, which increase fatty acid oxidation (FAO) required for the cell metabolism in radioresistant breast cancer cells and radiation‐derived breast CSCs.^233^ SCD1 (stearoyl‐CoA desaturase 1) is another functional downstream molecule of PPARα, and the activation of the PPARα‐SCD1 axis is important to the maintenance of CSCs of HCC.^234^ PPARα, on the other hand, is considered a downstream molecule of lipid droplet‐derived signaling, which was highly abundant in pancreatic and colorectal CSCs than non‐CSCs. The inhibition of PPARα decreases stemness characteristics of pancreatic and colorectal CSCs.^235^ Likewise, PPARδ is involved in the maintenance of HSCs by regulating the FAO pathway. The inhibition of PPAR‐δ or mitochondrial FAO reduced the stemness of HSCs, whereas PPAR‐δ agonists enhanced HSC maintenance.^236^

On the contrary, PPARγ is considered a tumor suppressor that reduces the CD49^high^/CD24^+^ mesenchymal stem cells (MSCs) and inhibits tumor angiogenesis of breast cancer.^237^ The existence of quiescent LSCs may contribute to treatment failure in CML patients. PPARγ agonist glitazones decrease the expression of STAT5 and its downstream targets HIF2α and CITED2, two key genes maintaining the quiescence and stemness of CML LSCs.^238^ PPARγ activation decreases the stem cell‐like characteristics of bladder CSCs and accelerates the differentiation of adipocytes.^239^ PPARγ also induces the differentiation in osteosarcoma stem cells and melanoma cells by suppressing the transcriptional activity of YAP.^240^, ^241^ Notably, PTEN is a target gene of PPARγ activation, which in turn blocks the PI3K/Akt/mTOR pathway and prevents the self‐renewal, tumorigenicity, and metastasis of cervical, hepatic, and glioblastoma CSCs.^242^, ^243^

### 3.2 Extrinsic signaling pathways that regulate CSCs

The “seed and soil” theory was first brought up in the 19th century, which describes the metastasis of tumor cells to sites with a favorable microenvironment.^244^ In this theory, the “seed” refers to the metastatic tumor cells, and “fertile soil” refers to the sites with a microenvironment that favors tumor colonization and growth. In accordance with the “seed and soil” theory, it is widely accepted that CSCs dwell in such “soil,” a specific tumor microenvironment (TME) composed of stroma, immune cells, microvessels, and external regulating signals.^245^ This system provides CSCs with a conductive environment via the action of paracrine factors or direct contact with immune cells.^8^, ^246^

#### 3.2.1 Vascular microenvironments that regulate CSCs

The theory of tumor vascular microenvironment dates back to the 1940s when glioblastoma cells were found to grow into the blood vessels‐enriched sites.^247^ The multilineage differentiation capacity of CSCs may allow them to take part in tumor angiogenesis or forming the vascular mimicry (VM) in the TME. Recent research has shed light on the relationship between CSCs and the vascular microenvironment. A typical example is glioblastoma CSC, where the expression of surface marker Nestin is positively related to microvessel density.^248^ The vascular endothelium of glioblastoma has similar genomic alterations to CSCs.^21^ Neural stem cells cocultured with epithelial cells demonstrated increased self‐renewal and impaired differentiation ability via paracrine signaling, including the Notch pathway and the chemokine axis CXCL12/CXCR4.^249^, ^250^, ^251^

Recently, the CSC surface marker CD44 was reported to promote the VM generation in oral squamous cell carcinoma.^252^ The CD44/c‐Met signaling has also been identified as the key regulator for VM in Ewing sarcoma and breast cancers.^253^ The presence of VM is also associated with ALDH1 expression in breast cancer.^254^, ^255^ ALDH^+^ TNBC cells isolated from FACS initiated VM on matrigel.^256^ The increased expression level of VM‐related genes, such as MMP‐2 and MMP‐9, was observed in CD133^+^ breast cancer cells.^257^

The regulation of CSC phenotype by edothelial cells (ECs) can be based on the secretion of soluble factors by ECs.^248^ In acute leukemia, bone marrow stromal cells derived from CD133^+^/CD34^+^ stem cells secrete IGF‐1, leading to the formation of capillary‐like structures.^258^ Shh is a soluble factor secreted by ECs, which enhances CSC properties and stimulates the Hh signaling.^259^, ^260^, ^261^ Hh signaling facilitates the acquisition of CSC self‐renewal in thyroid cancer, via regulating Snail expression.^260^ Interestingly, CD133^+^ GSCs were identified in areas surrounding Shh‐expressing ECs. The depletion of Shh in ECs prevents of promoting effect of ECs on CSC‐like phenotype maintenance.^259^

In addition to HH signaling, Notch signal cascades also take an active part in the EC‐mediated regulation of CSCs.^262^, ^263^, ^264^, ^265^ In CRCs, the promotion of CSC phenotypes by ECs is dependent on Notch signaling and independent of Shh or Wnt signaling. The knockout of Jagged‐1, a Notch signaling element, in EC impairs its angiocrine effect.^263^ The nitric oxide derived from ECs is able to trigger Notch signaling, leading to increased stemness of GSCs and glioma initiation in mice.^264^ Furthermore, in SHH‐driven medulloblastomas, the EC‐induced promotion of CSC characteristics additionally requires PI3K/AKT/mTOR signals.^266^

#### 3.2.2 Hypoxic microenvironments that regulate CSCs

Hypoxia is a common hallmark of TME in solid tumors.^267^, ^268^ In solid tumors, the rapidly proliferating tumor cells require a high level of oxygen to meet the expanding demands, resulting in relative hypoxia.^269^, ^270^ In this sense, an extreme hypoxic environment appears as the natural selection for tumor cells, where aggressive CSCs are more likely to survive and proliferate.^271^, ^272^ It is thus not surprising that CSCs are more resistant to conventional cancer therapies.^273^

Hypoxia leads to the acquisition of CSC phenotypes in breast tumors, which is primarily mediated by HIFs.^274^ HIFs (HIF‐1, HIF‐2, and HIF‐3) are key sensors of intracellular oxygen alterations and modulate the transcription of multiple genes at low oxygen levels.^275^, ^276^, ^277^ The Notch signaling is a key regulating pathway for hypoxia response, which can be activated by HIF‐1α and HIF‐2α for the maintenance of CSC stemness.^278^, ^279^ For instance, the HIF‐2α‐mediated Notch pathway activation promotes the phenotypic transformation of breast cancer cells into breast CSCs and cell resistance to paclitaxel treatment.^107^ Likewise, hypoxia‐induced AKT activation contributes to gemcitabine‐induced stemness of pancreatic cancer cells by enhancing downstream Notch1 activity.^280^ Another downstream element of PI3K/AKT signaling, mTOR, regulates HIF‐1α activity via phosphorylation of p70 S6Kinase (S6K).^281^ However, the inactive state of mTOR also facilitates the maintenance of CSC characteristics, which explains the suboptimal efficacy of mTOR inhibitors in clinical evaluation.^222^ The agonist of PTEN, a negative regulator of the PI3K/AKT pathway, provides a new clue to inhibit HIF‐1α activities and thus reduce CSC stemness.^282^

Fibroblasts are major components of tumor stroma, which are able to produce various extracellular matrix proteins and growth factors, such as TGF‐β.^283^ Hypoxia induces the upregulation in TGF‐β3 expression by promoting the binding of HIF‐1 to the TGF‐β3 gene promoter.^284^ It has also been reported that hypoxia increased TGF‐β1 expression in MSCs.^285^ The hypoxia‐induced secretion of TGF‐β1 by MSCs in turn enhances tumor progression,^286^ potentially by promoting the stabilization of HIFs.^8^ Thus, the concomitant inhibition of HIF‐1α and TGF‐β delays tumor initiation and blocks the activity of CSCs.^287^

Fibroblasts‐directed CSC reprogramming includes the stimulation of COX‐2 and nuclear factor of κB (NF‐κB).^8^ On one hand, hypoxia‐mediated downregulation of dual specificity phosphatase 2 (DUSP2) upregulates COX‐2, leading to increased cancer stemness.^288^ On the other hand, HIF‐1α induces COX‐2 expression, which in turn upregulates HIF‐2α expression and enhances treatment resistance of cancer cells.^289^

#### 3.2.3 Immune cells that regulate CSCs

**FIGURE 2:**
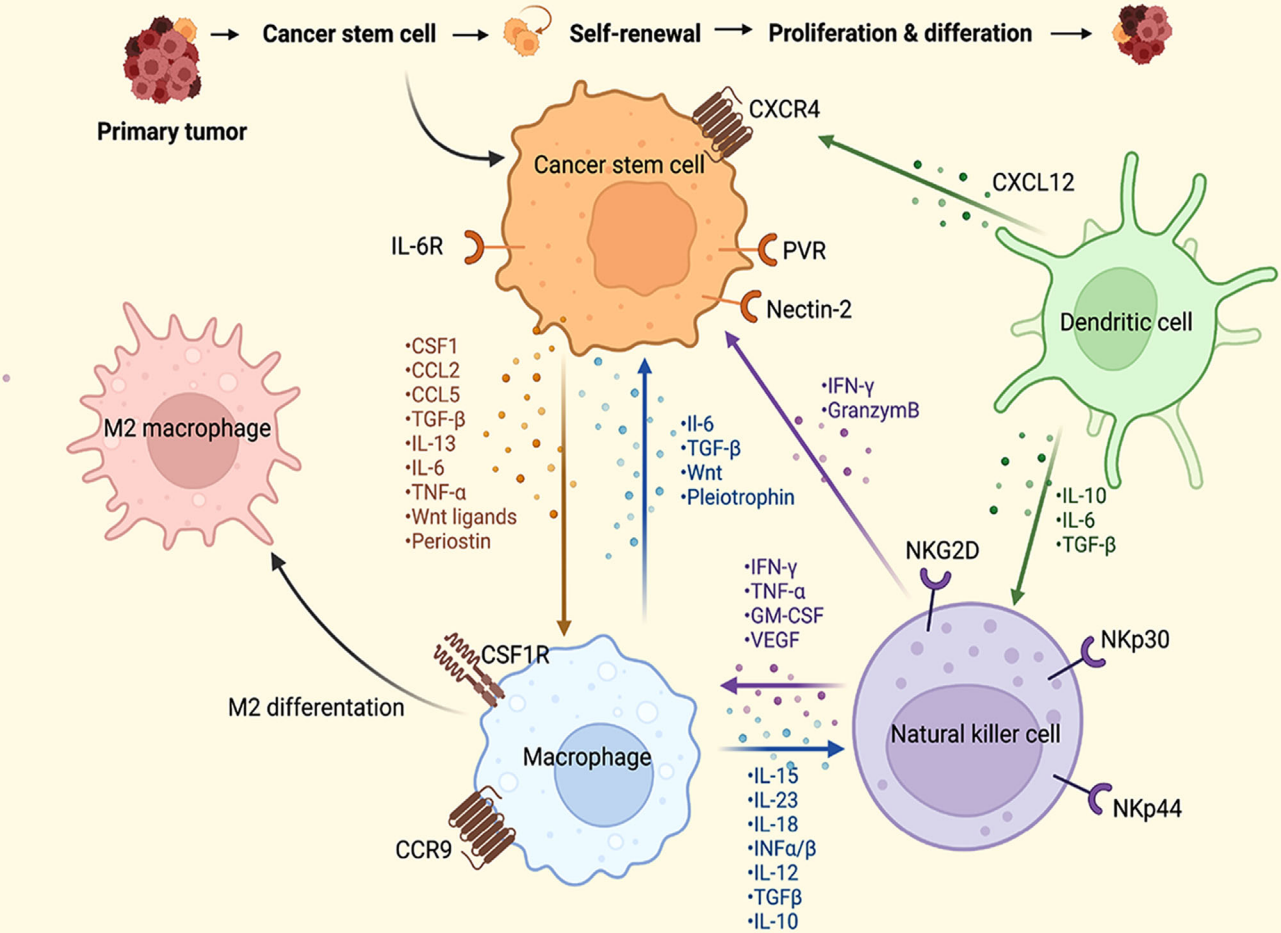
The crosstalk between cancer stem cells (CSCs) and immune cells in the CSC niche via soluble mediators or juxtacrine signals, which regulate CSC stemness. (Figures built with biorender.com)

As immune evasion and CSCs both substantially contribute to tumor progression, it is widely accepted that there is potential crosstalk between CSCs and immune cells in the TME. A significantly high stemness signature was identified in 21 solid malignancies with a poor immunogenic response.^290^ It is thus of paramount importance to elucidate the CSC‐immune cell interactions in cancer, which will facilitate the identification of immunotherapies to eliminate tumor‐promoting CSCs. Figure 2 presents the crosstalk between CSCs and immune cells in the CSC niche, which regulates CSC stemness.

##### 3.2.3.1 Tumor‐associated macrophage

The critical role of CSCs in monocyte recruitment to tumor sites has been well established as various protumorigenic macrophage factors were increased in supernatant collected from CSC sphere culture, including IL‐13, TGF‐β, and WNT‐induced signaling protein 1.^291^, ^292^, ^293^ Incubation of macrophages with such sphere culture leads to macrophage polarization toward an immunosuppressive phenotype.^294^, ^295^, ^296^

On the other hand, TAMs in turn influence CSC phenotypes by secreting soluble mediators, such as IL‐6, TGF‐β, and WNT ligands, or through juxtacrine signaling.^297^, ^298^ The direct interactions of CSCs with TAMs activates NF‐κB in CSCs, which stimulates the secretion of cytokines to sustain the stem cell state of breast CSCs.^299^ In the pleiotrophin (PTN)‐PTPRZ1 paracrine signaling, which supports glioma progression, PTN released by TAMs binds to its receptor PTPRZ1 on GSCs, suggesting the significance of TAMs as important components of the CSC niche.^300^

IL‐6 produced by TAMs promotes the expansion of hepatic CSCs, and the inhibition of IL‐6 with tocilizumab prevents TAM‐stimulated generation of CD44^+^ cells.^301^ In breast cancer, TAM‐produced IL‐6 induces and maintains the CSC characteristics through STAT3.^302^ In addition, STAT3 is a transcription factor that could also modulate CSC maintenance in an IL‐6‐independent manner. For instance, the self‐renewal and tumorigenicity of bladder CSCs are regulated by the KMT1A‐GATA3‐STAT3 circuit, which is independent of IL‐6.^303^ The STAT3 blockade decreased the expression of PD‐L1 on CD44^+^ cells in squamous cell carcinoma of the head and neck, a well‐characterized cell population with CSC characteristics, resuming T‐cell‐mediated immunity.^304^ These results further justify the development of IL‐6^‐^ or STAT3‐targeting strategies in cancer treatment.

##### 3.2.3.2 Natural killer cell

Though CSCs were previously believed as less immunogenic than non‐CSCs due to decreased MHC class‐I (MHC I) expression,^305^, ^306^ growing evidence now suggests that CSCs are preferentially susceptible to natural killer (NK) cell activities.^307^, ^308^ This vulnerability may be attributed to the activated natural cytotoxicity receptors, particularly NKp30 and NKp44. Though glioblastoma CSCs express deficient MHC I molecules, various ligands that activate NK cell receptors were found on these CSCs, such as PVR and Nectin‐2.^308^ Interestingly, CSCs were resistant to NK cells freshly isolated from tumor specimen, but were sensitive to the activities of both allogeneic and autologous IL‐2 or IL‐15‐activated NK cells.^308^ In melanoma, both CD133^‐^ and CD133^+^ subpopulations are susceptible to the cytotoxicity of IL‐2‐activated allogeneic NK cells.^309^ Likewise, the increased sensitivity of breast CSCs to IL‐2 or IL‐15‐activated NK cells, which is potentially mediated by the upregulation of NKG2D ligands ULBP1, ULBP2, and MICA on CD44^+^CD24^−^ breast CSCs.^310^ Similar results were observed in ovarian cancer^311^ and CCR7^+^ melanoma.^312^

Notably, an increased frequency of CSCs is often observed following cytotoxic treatments for primary cancers.^313^, ^314^ A study reported a novel mechanism for the immune escape of breast CSCs from NK cell attack, due to decreased expression of ligands that stimulate NKG2D.^315^ The upregulation of the NKG2D stress ligands MICA/B on surviving CSCs following cytotoxic treatments, such as radiotherapy, sensitizes CSCs to NK cell killing. NK cells were recruited to the tumor‐adjacent areas but lost their cytotoxic efficacy in breast tumors due to the altered ligand expression ligands on radioresistant breast CSCs.^316^ This evidence further provides a rationale for combining the NK cell‐stimulating factors with conventional therapies.

##### 3.2.3.3 Cancer‐associated fibroblasts and MSCs

The oncogenic effect of cancer‐associated fibroblasts (CAFs) is mostly based on their secretion of a number of paracrine factors, including proinflammatory cytokines, chemokines, prostaglandins, growth factors, and proteases, which collectively promote tumor growth, angiogenesis, and invasion.^317^, ^318^, ^319^ CAFs are also believed to create an immunosuppressive TME by potentiating regulatory T cells,^320^ or induce M2‑polarized macrophages.^321^ Moreover, CAFs‐derived exosomes lead to treatment resistance of cancer.^322^, ^323^

Notably, one of the key mechanisms for the CAFs‐mediated tumor promotion is based on their regulation of CSC stemness.^324^ The paracrine factors produced by specific CAF subpopulations accelerate the transformation of cancer cells into CSCs and help maintain the stemness properties of existing CSCs.^325^ Under the cell stimuli, such as chemotherapy, CAFs acquire a senescence‐like secretory phenotype, and their secretion of prostemness chemokines is further increased, resulting in CSC‐associated chemoresistance.^326^

Both resident and recruited MSCs within TME can acquire CAF‐like phenotypes, suggesting that CAFs can be derived from MSC transformation.^327^ In pancreatic ductal adenocarcinoma (PDAC) and gastric cancer models, bone marrow‐derived MSCs are recruited to TME in a TGF‐β and CXCL‐12‐dependent manner and differentiate into CAFs.^328^ This transformation may be attributed to tumor‐secreted factors, such as the TGF‐β, which activate MSCs into CAFs, further enhancing the cell heterogenicity of the CSC microenvironment.^329^ MSCs are stromal cells with multipotent differentiation abilities and can migrate to tumor sites and promote tumor EMT via the secretion of various factors.^330^ For example, in gastric cancer, MSCs secret VEGF, macrophage inflammatory protein‐2, TGF‐β1, and the proinflammatory cytokines interleukin IL‐6 and IL‐8, which collectively facilitate tumor growth and angiogenesis.^331^

## 4 THERAPIES TARGETING SIGNALING PATHWAYS OF CSC‐s

Given that CSCs are a major contributing factor to progressive phenotypes of cancer, targeting CSCs in the tumor now appears as a promising strategy against cancer. Numerous efforts have been undertaken these years to identify such therapies, such as kinase inhibitors and antibodies that block CSC‐associated signaling pathway elements, and some of these approaches have already entered the clinical phase.^332^, ^333^ The ongoing and completed clinical trials on therapies targeting signaling pathways of CSCs are presented in Table 2. Immunotherapies targeting CSCs include MHC‐restricted killing, such as checkpoint inhibitors, and MHC‐unrestricted killing, such as the chimeric antigen receptor (CAR) T‐cell approach.^334^, ^335^

**TABLE 2:** Ongoing and completed clinical trials on therapies targeting signaling pathways of CSCs

Agents (targets)	Condition	Cotherapy	Phase	NCT number
Gamma‐secretase inhibitors (GSIs)				
RO4929097 (Notch, Aβ40, secretase)	Metastatic pancreas cancer		II	NCT01232829
	Advanced solid tumors		I	NCT01145456
	Advanced solid tumors	Cediranib maleate	I	NCT01131234
	Advanced solid tumors		I	NCT01096355
	Refractory NSCLC		II	NCT01070927
	Metastatic epithelial ovarian cancer, fallopian tube cancer, or primary peritoneal cancer		II	NCT01175343
	Advanced sarcoma		I/II	NCT01154452
	Advanced solid tumors	Capecitabine	I	NCT01158274
	Advanced renal cell carcinoma after VEGF/VEGFR therapy failure		II	NCT01141569
	Advanced solid tumors		I	NCT0121862
	Advanced solid tumors	Temsirolimus	I	NCT01198184
	Malignant glioma	Temozolomide and radiation therapy	I	NCT01119599
	Metastatic melanoma	Cisplatin, vinblastine, and temozolomide	I/II	NCT01196416
	Metastatic colorectal cancer		II	NCT01116687
PF‐03084014 (secretase)	Desmoid tumors		II	NCT01981551
	Advanced cancer and leukemia		I	NCT00878189
MK‐0752 (secretase)	Advanced breast cancer	Docetaxel	I/II	NCT00645333
	Early‐stage breast cancer	Tamoxifen/letrozole	IV	NCT00756717
	Pancreatic cancer	Gemcitabine hydrochloride	I	NCT01098344
	Advanced cancer	Ridaforolimus	I	NCT01295632
	Advanced breast cancer		I	NCT00106145
Pan‐Notch small molecule inhibitor				
BMS‐906024 (γ‐Secretase and Notch)	Advanced solid tumors		I	NCT01292655
	Advanced solid tumors	Chemotherapy	I	NCT01653470
	Acute T‐cell lymphoblastic leukemia or T‐cell lymphoblastic lymphoma		I	NCT01363817
CB‐103 (Notch)	Luminal advanced breast cancer	Nonsteroidal aromatase inhibitor	II	NCT04714619
	Advanced solid tumors and hematological malignancies		I/II	NCT03422679
Monoclonal antibodies (mAbs) targeting Notch				
MEDI0639 (Dll4)	Advanced solid tumors		I	NCT01577745
SIBP‐03 (HER3)	Advanced solid tumors		I	NCT05203601
OMP‐52M51 (Notch1)	Metastatic colorectal cancer		I	NCT03031691
	Advanced solid tumors		I	NCT01778439
	Refractory lymphoid malignancies		I	NCT01703572
	Adenoid cystic carcinoma		NA	NCT02662608
OMP‐59R5 (Notch 2/3)	Stage IV pancreatic cancer	Nab‐paclitaxel and gemcitabine	I/II	NCT01647828
	Advanced solid tumors		I	NCT01277146
SMO inhibitor				
BMS‐833923 (SMO)	Chronic myeloid leukemia		I/II	NCT01218477
LEQ506 (SMO)	Advanced solid tumors		I	NCT01106508
Vismodegib (SMO)	Prostate cancer		I	NCT02115828
	Metastatic colorectal cancer	Chemotherapy	II	NCT00636610
	Ovarian cancer		II	NCT00739661
	Keratocystic odontogenic tumor		II	NCT02366312
	Advanced pancreatic cancer	Gemcitabine hydrochloride	I/II	NCT01064622
			II	NCT01195415, NCT01088815
	Advanced Solid Tumors		I	NCT01546519, NCT03878524, NCT00878163, NCT00607724, NCT01209143, NCT00968981, NCT01537107
			II	NCT05159245, NCT05238831, NCT05159245, NCT02091141, NCT00959647
	Basal cell carcinoma		I	NCT01631331, NCT02639117, NCT03158389
			II	NCT01543581, NCT03035188, NCT00833417, NCT01700049, NCT02667574, NCT01201915, NCT02371967, NCT01367665, NCT01556009, NCT01815840, NCT04416516
			IV	NCT03610022, NCT02436408
	Head/neck basal cell carcinoma	Radiation therapy	II	NCT01835626
	Advanced gastric adenocarcinoma		II	NCT03052478
	Advanced stomach cancer or gastroesophageal junction cancer	Chemotherapy	II	NCT00982592
	Basal cell skin cancer		I/II	NCT02690948
	Small cell lung carcinoma	Cisplatin and etoposide	II	NCT00887159
	Advanced sarcoma		I/II	NCT01154452
	Multiple myeloma		I	NCT01330173
	Recurrent glioblastoma		II	NCT00980343
	Advanced urothelial carcinoma		II	NCT02788201
	Advanced malignancies		II	NCT02465060
	Advanced chondrosarcomas		II	NCT01267955
	Refractory medulloblastoma		II	NCT01239316, NCT00939484, NCT01878617
			I	NCT00822458
	Progressive meningiomas		II	NCT02523014
Sonidegib (SMO)	Basal cell carcinoma		NA	NCT01529450
			II	NCT00961896, NCT03534947, NCT01327053, NCT04806646, NCT01350115, NCT00961896
	Advanced solid tumors		I	NCT00880308, NCT01208831,
		Paclitaxel	I	NCT01954355
		BKM120	I	NCT01576666
		Pembrolizumab	I	NCT04007744
	Myeloid leukemia	Nilotinib	I	NCT01456676
	Prostate cancer		I	NCT02111187
	Triple‐negative (TN) advanced breast cancer (ABC)	Docetaxel	I	NCT02027376
	Recurrent ovarian cancer	Paclitaxel	I	NCT02195973
	Extensive stage small cell lung cancer (ES‐SCLC)	Etoposide and cisplatin	I	NCT01579929
	Pancreatic cancer	Gemcitabine and nab paclitaxel	I/II	NCT02358161
		Gemcitabine	I	NCT01487785
		Chemotherapy	I	NCT01485744
	Esophageal cancer	Everolimus	I	NCT02138929
	Myeloid malignancies	Azacitidine	I	NCT02129101
	Recurrent brain tumors		I	NCT03434262
	Multiple myeloma		II	NCT02086552
	Medulloblastoma		II	NCT01708174, NCT04402073
	Hepatocellular carcinoma		I	NCT02151864
	Acute leukemias		II	NCT01826214
	Chronic myelogenous leukemia	Nilotinib	I	NCT01456676
Glasdegib (SMO)	Acute myeloid leukemia, chronic myelomonocytic leukemia	Azacitidine	I	NCT02367456
	Acute myeloid leukemia		II	NCT01546038, NCT01841333, NCT03226418
	Soft tissue sarcoma		III	NCT03784014
	Glioblastoma	Temozolomide and radiotherapy	I/II	NCT03529448
Inhibitors of WNT pathway elements				
DKN‐01 (DKK1)	Advanced biliary tract cancer	Nivolumab	II	NCT04057365
	Prostate cancer	Docetaxel	I/II	NCT03837353
	Multiple myeloma or advanced solid tumors		I	NCT01457417
	Epithelial endometrial or epithelial ovarian cancer	Paclitaxel	II	NCT03395080
	Gastric or gastroesophageal cancer	Tislelizumab ± chemotherapy	II	NCT04363801
		Paclitaxel or pembrolizumab	I	NCT02013154
	Advanced liver cancer		I/II	NCT03645980
	Cancer of hepatic biliary system or gallbladder	Gemcitabine + cisplatin	I	NCT02375880
	Multiple myeloma	Lenalidomide/dexamethasone	I	NCT01711671
Vantictumab (FZD receptors)	Metastatic breast cancer		I	NCT01973309
	Pancreatic cancer	Nab‐paclitaxel and gemcitabine	I	NCT02005315
	NSCLC	Docetaxel	I	NCT01957007
Cirmtuzumab (ROR1)	Metastatic castration‐resistant prostate cancer		II	NCT05156905
	Breast cancer	Cirmtuzumab + paclitaxel	I	NCT02776917
	B‐cell lymphoid malignancies	Ibrutinib	I/II	NCT03088878
	Refractory chronic lymphocytic leukemia		I	NCT02222688
			II	NCT04501939

Clinical trial data sources: clinicaltrials.gov.

### 4.1 Targeting Notch signaling

Tumors with NOTCH1 mutations represent a distinct tumor phenotype with increased activation in Notch1 signaling. NOTCH1‐mutant tumors are often associated with metastasis, poor prognosis, and potential responsiveness to brontictuzumab.^336^

#### 4.1.1 Gamma‐secretase inhibitors

A number of Notch‐pathway inhibitors have been developed with different action mechanisms, some of which are currently under clinical evaluation. Gamma‐secretase inhibitors (GSIs) have long been identified as a large family of Notch‐targeted small molecule inhibitors, by blocking the proteolytic cleavage of Notch receptors. GSI PF‐03084014 inhibited tumor growth in a mouse xenograft model of T‐cell acute lymphoblastic leukemia.^337^, ^338^ GSI MRK‐003 works synergistically with trastuzumab in HER2‐positive breast cancer mouse model.^339^ Likewise in NSCLC models, BMS‐906024 has demonstrated potent antitumor efficacy in combination with chemotherapies, such as cisplatin, paclitaxel, docetaxel, and target therapies, such as crizotinib.^340^, ^341^

The ability of GSIs to block Notch signaling and subsequently reduce CSC burden in preclinical studies has spurred clinical assessment of GSIs in clinical trials. A well‐studied GSI RO4929097 substantially reduced the expression level of stem cell markers on primary melanoma cells and inhibited tumor formation in melanoma xenograft transplants.^342^ In a phase II trial, RO4929097 was well tolerated in patients previously treated with PDA, with 25% of patients achieving stable disease.^343^ In a phase I trial, four of 24 melanoma patients were reported with clinical benefits from RO4929097 treatments, with one patient achieving a complete response.^344^ This encouraged the following phase II trial of RO4929097 in patients with metastatic melanoma with monotherapy.^345^ Besides, the combinational treatment of GSIs with other cancer treatments further improved clinical outcomes. One such example is the combination of RO4929097 with bevacizumab in patients with malignant gliomas.^346^ These clinical results suggest that GSIs can effectively cross the blood‐brain barrier and reach therapeutic concentrations at tumor sites. However, RO4929097 monotherapy displayed minimal inhibition of neurosphere formation in recurrent glioblastoma samples.^347^ Similarly, in metastatic CRCs, the antitumor activity of GSIs, including RO4929097,^344^ LY900009,^348^ MK‐0752,^349^ and BMS‐986115^350^ as monotherapies, is suboptimal.^351^ A recent phase Ib/II trial evaluated the treatment combination of RO4929097 with vismodegib, an HH inhibitor, in advanced sarcoma, providing a rationale for the synergy of GSIs in this patient population.^352^

PF‐03084014 and MK‐0752 are two GSIs frequently used in clinical studies, both of which have been used to treat advanced‐stage solid tumors but failed to reach evident clinical efficacy in patients with lung cancer, breast cancer, or pancreatic cancer as monotherapies.^353^ As such, these failures further encouraged combinatorial regimens of GSIs with other anticancer therapies, as evidenced by the fact that PF‐03084014 enhanced the antitumor effect of DOX in prostate cancer stem‐like cells.^354^ Moreover, the concomitant use of RO4929097 and cediranib has prolonged disease stabilization in 11 out of 20 patients with advanced solid tumors.^355^

The most common dose‐limiting toxicities (DLTs) of GSIs occur in the gastrointestinal system, with secretory diarrhea accounting for 30–60% of all reported DLTs in cancer patients and grade ≥3 diarrhea accounting for around 11%. This may be explained by the fact that inhibition of Notch1 and Notch2 prevents the proliferation of crypt progenitors leading to goblet‐cell metaplasia of the small‐intestinal epithelium.^356^ The addiction to glucocorticoids and antiestrogens in the GSI treatment regimens significantly relieved GSI‐induced gastrointestinal toxicities.^338^, ^357^ Hypophosphatemia is another GSIs‐induced toxicity, which is potentially caused by abnormal gastrointestinal function and can be relieved by oral administration of phosphate replacement.^358^

#### 4.1.2 Pan‐Notch small molecule inhibitor

Though Notch1 is the most common activated oncogene in tumors, the coexpression of Notch1 and Notch4 is frequently observed in breast cancer. Moreover, accumulating evidence suggests the Notch3‐mediated progression of cancer. These results collectively reveal the requirement for pan‐Notch inhibition to achieve a broader spectrum of antitumor efficacy. A previous study evaluated the structure–activity relationships in a series of (2‐oxo‐1,4‐benzodiazepin‐3‐yl)‐succinamides as pan‐Notch inhibitors.^359^ Among these GSIs, MS‐906024 displayed the broadest spectrum efficacy in multiple in‐vivo tumor models and thus advanced into clinical trials (NCT01292655).^360^ BMS‐906024 sensitizes NSCLC to paclitaxel treatment, and patients with wild‐type KRAS and BRAF tumors may have improved response to the BMS‐906024 ^+^ paclitaxel combination.^340^ It was later reported that BMS‐906024 significantly enhanced the delay in NSCLC tumor spheroid growth delay caused by etoposide and crizotinib, and the most prominent delay in spheroid growth was observed in cells treated with BMS‐906024 + chemoradiation triple combination.^341^

CB‐103 is an oral pan‐Notch inhibitor that specifically targets protein–protein interaction by suppressing the Notch transcriptional complex.^361^ Another small molecule inhibitor, IMR‐1, inhibits the recruitment of MAML1 to the notch transcriptional complex, thereby preventing its activation.^362^ This approach has the advantage of acting downstream of aberrant Notch receptor activation by blocking the assembly of the transcription complex and thereby inhibiting the expression of Notch target genes. A phase I/IIa study is under way to investigate the safety and efficacy of CB‐103 in patients with advanced solid tumors and hematological malignancies (NCT03422679). In a phase II trial, patients with advanced breast cancer will receive the combinational treatment of CB‐103 with NSAI therapy (letrozole or anastrozole, continuing prior therapy) to evaluate the efficacy (NCT04714619).

#### 4.1.3 Monoclonal antibodies targeting Notch signaling

Brontictuzumab (OMP‐52M51) is a humanized monoclonal antibody (mAb) that selectively targets Notch1 juxtamembrane negative regulatory region and thus inhibits Notch signaling. In a phase II trial of brontictuzumab, six of total 36 (17%) patients with refractory solid tumors demonstrated clinical benefits, with four patients displaying prolonged disease stabilization (NCT01778439).^363^ A functional assay evaluated the efficacy of brontictuzumab in a series of glioma stem‐like cell models, supporting brontictuzumab as a promising drug candidate for CNS tumors.^364^

Tarextumab (OMP‐59R5) is a human IgG2 antibody with inhibition on both Notch2 and Notch3 and has shown encouraging antitumor efficacy in small cell lung cancer (SCLC). An overall response rate (ORR) of 84% was reported in phase I/II trial when patients received the combination of tarextumab with etoposide and platinum‐based therapies. Meanwhile, tarextumab leads to potent inhibition in Notch signaling, and tarextumab‐induced diarrhea was dose‐limiting above 2.5 mg weekly and 7.5 mg/kg every third week (NCT01277146). The triple combination of tarextumab in combination with gemcitabine plus nab‐paclitaxel resulted in increased inhibition of tumor growth and tumor‐initiating cell frequency compared with the combination of tarextumab with gemcitabine alone.^365^ However, a randomized phase II trial found that the addition of tarextumab to nab‐paclitaxel and gemcitabine failed to induce a prolonged overall survival (OS), progression‐free survival (PFS), or ORR in patients with metastatic PDAC.^366^ The specific role of Notch signaling in PDAC remains unclear with evidence supporting both its oncogenic and tumor‐suppressive roles.^366^ Further research using individual Notch inhibitors and agonists may facilitate the clinical evaluation of Notch‐targeting agents in pancreatic cancer.

### 4.2 Targeting HH signaling

The pharmacological inhibition targeting the HH pathway in cancer is an active research field, some of which have received regulatory approvals. Major HH pathway antagonists investigated so far include SMO inhibitors and GLI inhibitors.^367^

#### 4.2.1 SMO inhibitor

Two SMO antagonists, vismodegib and sonidegib, have been approved by the Food and Drug Administration (FDA) for the treatment of advanced basal cell carcinoma (BCC). Vismodegib (GDC‐0449) was first granted approval following success in clinical trials in 2012, where the independently assessed response rate was 30% in 33 patients with metastatic BCC (NCT00833417).^368^ In the subsequent phase II trial, a total number of 1215 patients with advanced BCC were treated with vismodegib (NCT01367665). The response rate of patients with metastatic disease was 36.9% and that in patients with locally advanced disease was 68.5% 29073584. However, no benefits were obtained from the additional use of vismodegib in standard treatment regimens for metastatic CRC,^368^ PDAC,^369^ gastric cancer, and gastroesophageal junction cancer.^370^ Besides, the combinations of erlotinib or chemotherapy with vismodegib were well tolerated but induced no improved outcome in metastatic PDAC or PDA, respectively.^371^ Vismodegib also failed to deliver clinical benefits compared with placebo as maintenance therapy for ovarian cancer patients.^372^ Vismodegib improved PFS in patients with SHH‐subtype medulloblastoma but not in those with non‐SHH disease subtypes.^373^, ^374^, ^375^, ^376^ Recent evidence, however, suggested that the addition of vismodegib to TMZ did not improve PFS even in SHH refractory medulloblastoma.^377^ A recent phase II trial suggested that the histopathologic subtypes of BCC had no significant impact on patient response to vismodegib.^378^

Sonidegib (LDE225) was approved by FDA in 2015 based on the promising results from a randomized phase II study, where sonidegib at 200 and 800 mg daily induced similar ORR (58% vs. 44%) in patients with locally advanced BCC.^135^ In the following analyses, sonidegib demonstrated long‐term efficacy and safety profile (30^379^ and 42 months^380^) in patients with advanced BCC. A meta‐analysis showed that ORRs of vismodegib and sonidegib were comparable in locally advanced BCC (69% vs. 57% respectively), whereas the complete response rates of the two drugs were different (31% vs. 3% respectively).^381^ Likewise, BCC patients resistant to vismodegib similarly developed resistance with sonidegib.^382^ In both mouse models and phase I clinical trial of TNBC, sonidegib decreased CSC markers expression and sensitizes cancer cells to docetaxel chemotherapy.^383^

Another key SMO inhibitor, glasdegib (PF‐04449913), received FDA approval in 2018 as a combination partner for cytarabine for the treatment of AML. The addiction of glasdegib to low‐dose cytarabine (LDAC) increased the median OS of newly diagnosed AML patients from 4.9 to 8.8 months.^384^ Glasdegib + LDAC continued to induce long‐term survival benefits in patients with AML, especially with secondary AML (NCT01546038).^385^ In phase II clinical trial, 46.4% of patients achieved CR after glasdegib + cytarabine and daunorubicin treatment (NCT01546038).^386^ The subsequent phase III trial of glasdegib in combination with chemotherapy (7 + 3 schedules) to treat AML patients is currently under way (NCT03416179).^384^

#### 4.2.2 Inhibitors of GLI transcriptional activity

GLI‐mediated transcription constitutes the final step of the HH pathway and the inhibition of GLI transcription factors is thus a promising strategy that reduces tumor cell proliferation.^387^ Inhibitors of GLI‐mediated transcription, such as GANT58 and GANT61, were first designed to overcome tumor resistance to SMO inhibitors. A wide breadth of literature has described the antitumor activity of these agents in various cancers, including NSCLC, breast cancer, prostate cancer, and rhabdomyosarcoma.^388^, ^389^, ^390^, ^391^, ^392^ However, neither of these two agents have advanced into clinical trials.

Arsenic trioxide (ATO), an FDA‐approved drug widely accepted in treating acute promyelocytic leukemia, is also a potent inhibitor of GLI1 and GLI2 and inhibits cancer growth by blocking GLI transcription.^392^ With its inhibition of GLI transcription activities, ATO inhibits the viability and maintenance of CSCs derived from SCLC^393^ and pancreatic cancer.^394^ ATO also prevents osteosarcoma growth via DNA damage accumulation.^395^ In a phase II study, the concomitant use of ATO and itraconazole was tested in BCC patients who were resistant to SMO inhibitors. Significant alterations in mRNA levels of GLI1 were observed.^396^ Given that none of the participants had tumor shrinkage though they experienced SD for 3 months, continuous dosing was later recommended to achieve a better clinical response.^396^ Currently, multiple clinical trials of ATO, alone or in synergy with standard therapies in cancer patients, are under way.

### 4.3 Targeting Wnt signaling

The Wnt pathway inhibitor family is mainly comprised of agents targeting Wnt pathway molecules, Porcupine inhibitors that diminish the ability to secrete Wnt ligands, and inhibitors of downstream β‐catenin‐TCF‐LEF‐dependent transcription. Many of these agents have been extensively studied and are currently under clinical evaluation.

#### 4.3.1 Inhibitors of Wnt pathway elements

DKN‐01 is an IgG4 mAb targeting Dkk1 that suppresses canonical Wnt signaling via negative feedback.^397^ Some studies addressed the direct antitumor effects of DKK1 inhibition,^398^ whereas some recently reported its indirect antitumor effects via stimulation of immune responses in cancers, including ovarian cancer^399^ and prostate cancer.^400^ The murine version of DKN‐01 overcomes the DKK1‐mediated immune suppression and improves the efficacy of PD‐1 blockade.^401^ Similarly, inhibiting Wnt/β‐catenin signaling by DKN‐01 enhances the antitumor immune infiltration into tumors and improves the response of ovarian tumors to immune checkpoint inhibitors.^399^

Multiple clinical trials of DKN‐01 are now carried out across a wide range of cancer types. In a phase I trial, the combination of DKN‐01 with paclitaxel is well tolerated in patients with DKK1‐positive esophageal or gastroesophageal junction tumors (NCT02013154).^402^ In a following phase II trial, the combination of DKN‐01 with pembrolizumab was well tolerated in patients with a gastroesophageal junction or gastric cancer, and especially effective in anti‐PD‐1/PD‐L1‐naïve patients with DKK1^‐^ high tumors.^403^ A biomarker analysis revealed that DKN‐01 in combination with chemotherapies potentially led to reduced angiogenesis and inflammation markers in patients with biliary tract cancer (NCT02375880).^404^

Vantictumab is a fully human mAb that inhibits Wnt pathway signaling by targeting FZD1, 2, 5, 7, and 8 receptors. Vantictumab decreases the enrichment of CSCs in various tumor types, either alone or in synergy with a chemotherapeutic.^405^ A phase I study evaluated the combination of vantictumab with nab‐paclitaxel and gemcitabine in metastatic PDA patients. However, this trial was ultimately terminated due to bone‐related cytotoxicity.^406^ Another phase I study assessed the efficacy and safety of the combination of vantictumab with paclitaxel metastatic breast cancer and the further use of this combination was restricted by the frequently occurred fractures.^407^

Cirmtuzumab is a humanized mAb that inhibits the activity of ROR1, an oncoembryonic orphan receptor for Wnt5a in CSCs.^408^ The antitumor activities of cirmtuzumab are mostly documented in chronic lymphocytic leukemia (CLL), where it inhibits the activation of both NF‐κB and STAT3 in patients.^409^ Results from a phase I trial showed that cirmtuzumab is effective in suppressing tumor cell ROR1 signaling in CLL (NCT02222688).^410^ Targeting ROR1 with cirmtuzumab may also improve the response of breast cancer patients to chemotherapies.^411^ Cirmtuzumab could work synergistically with the Bruton tyrosine kinase inhibitor ibrutinib to treat patients with CLL or other ROR1^+^ B‐cell malignancies.^412^ Currently, a phase Ib/II study is under way to evaluate this combination in patients with CLL, small lymphocytic lymphoma, or mantle cell lymphoma (NCT03088878).

## 5 CSC‐DIRECTED IMMUNOTHERAPIES

As promising CSC‐directed immunotherapy, CSC‐based dendritic cell (DC) vaccines facilitate tumor cell recognition and eradication by potentiating antigen‐specific T‐cell responses against CSCs.^413^ The CSC‐specific T cells can also be produced by CSC priming. CSC lysate‐pulsed DCs stimulate CD8^+^ T cells, and the generated CSC‐specific T cells induce antitumor immunity by directly targeting CSCs in tumors.^414^, ^415^ Table 3 summarizes the ongoing and completed clinical trials on CSC‐directed immunotherapies. Bispecific antibodies (BiAbs) targeting CSC‐specific antigens represent another candidate for CSC‐directed immunotherapies. For instance, a BiAb composed of CD133 mAb monomer and a single chain of humanized muromonab‐CD3 targets CD133‐expressing tumor cells by arming activated T cells.^416^

**TABLE 3:** Ongoing and completed clinical trials on CSC‐directed immunotherapies

Immunotherapy	Condition	Phase	NCT number
Dendritic cell (DC) vaccines			
Cancer stem cells vaccine	Ovarian cancer	I/II	NCT02178670
Tumor lysate‐pulsed DC vaccine	High‐risk solid tumor	II	NCT00405327
Minor histocompatibility antigens (MiHA)‐loaded PD‐L‐silenced DC vaccination	Hematological malignancies	I/II	NCT02528682
Total tumor RNA (TTRNA)‐loaded‐DCs	Recurrent medulloblastoma and primitive neuroectodermal tumor	II	NCT01326104
CD34^+^‐derived DCs	Breast neoplasms	I	NCT00197522
Dendritic cell vaccine with mRNA from tumor stem cells	Glioblastoma	I/II	NCT00846456
Dendritic cell fusion vaccine	Multiple myeloma	II	NCT01067287
Autologous DCs with glioma stem‐like cells associated antigens	Glioblastoma	II	NCT01567202
Bispecific antibodies			
Anti‐CD3 × anti‐CD20 bispecific antibody‐armed activated T cells	Multiple myeloma and plasma cell neoplasm	I	NCT00938626
Elranatamab (B‐cell maturation antigen [BCMA] CD3‐targeted bispecific antibody)	Multiple myeloma	III	NCT05317416
Anti‐CD3 × anti‐CD20 bispecific antibody (CD20Bi)‐activated T cells (ATC)	Non‐Hodgkin lymphoma	I	NCT00244946

Clinical trial data sources: clinicaltrials.gov.

One of the most studied CSC‐directed immunotherapies that enter clinical trials is the CAR T‐cell transfer, based on the identification of CSC surface antigens by CAR T cells.^417^ Though a wide range of CSC‐related antigens are used to design CAR T‐cell therapies,^417^ CSC‐targeting CAR T cells to date have been approved by FDA. The largest concern about CAR T‐cell treatment could be its safety profile, cytokine release syndrome, and soluble tumor syndrome.^417^ The application of well‐characterized CSC markers in CAR T‐cell design is a promising approach to eliminate CSCs in many cancers, which, however, still requires further investigations to advance CAR T cells into the clinic.

A typical example of CAR T therapies is the CAR T cocktail immunotherapy composed of successive infusions of CART cells targeting epidermal growth factor receptor (EGFR) and CD133, which specifically target CSCs in cholangiocarcinoma (NCT01869166 and NCT02541370).^418^ CART‐133 cell therapy patients demonstrate promising antitumor activity. In HCC patients, CAR‐133 cell therapy demonstrated promising efficacy with manageable toxicity.^419^ This study also revealed potential biomarkers that predicted patient response to CART‐133 cells (NCT02541370). GBM CSCs are characterized as EGFRVIII^+^/CD133^+^ cells with self‐renewal as well as cancer initiation abilities.^420^ In the first clinical trial of EGFRVIII‐specific CAR T‐cell infusions, patients with EGFRVIII^+^ recurrent GBM did not obtain noticeable tumor regression according to MRI.^421^ Meanwhile, an additional study suggested that the CAR T‐EGFRVIII cell therapy failed to induce clinical benefits in patients with recurrent GBM.^422^ Ongoing and completed clinical trials on CSC‐directed CAR T‐cell therapy are presented in Table 4.

**TABLE 4:** Ongoing and completed clinical trials on CSC‐directed chimeric antigen receptor (CAR) T‐cell therapy

Chimeric antigen receptor (CAR) T cell	Condition	Phase	NCT number
CAR T‐cell therapy	Hematologic neoplasms	NA	NCT04691284
PSCA‐targeted CAR T cells (BPX‐601)	Advanced solid tumors	I/II	NCT02744287
Anti‐CD19 CAR T cells	B‐cell non‐Hodgkin lymphoma	I/II	NCT01318317
	B‐cell malignancies	I/II	NCT01475058, NCT01087294, NCT02659943
	Relapsed malignant lymphoma	I	NCT05239676
CD19CAR/virus‐specific T cells	CD19^+^ malignancies	I	NCT00840853
	CD19^+^ acute lymphoblastic leukemia or non‐Hodgkin lymphoma	I	NCT03768310
Ciltacabtagene autoleucel (BCMA‐directed CAR T)	Multiple myeloma	III	NCT04923893, NCT05257083
	B‐cell lymphoma	II	NCT04531046
Anti‐CD19 allo‐CAR T cells	Relapsed B‐cell malignancies	I	NCT04516551
Genetically modified T cells (CMV‐specific CD19‐CAR T cells)	B‐cell non‐Hodgkin lymphoma	I	NCT05432635
CD19^+^CD22 CAR‐T	B acute lymphoblastic leukemia	I	NCT04626726
CD19CAR‐CD28‐CD3zeta‐EGFRt‐expressing TCM‐enriched T cells	Non‐Hodgkin lymphoma	I	NCT01815749
	B‐cell non‐Hodgkin lymphoma	I	NCT02051257
CD19CAR‐CD28Z T cells	B‐cell lymphoblastic leukemia	I	NCT02050347
CAR T directed against CD19^+^ B cells	B‐cell non‐Hodgkin lymphoma	I	NCT01840566
Anti‐CD133‐CAR T cells	Advanced malignancies	I/II	NCT02541370
	Malignant gliomas	I	NCT03423992
Sarcoma‐specific CAR T cells	Sarcoma	I/II	NCT03356782
CD44v6‐specific CAR T cells	CD44v6‐positive cancers	I/II	NCT04427449
4SCAR T cells	Breast cancer	I/II	NCT04430595

Clinical trial data sources: clinicaltrials.gov.

The CSC characteristics are associated with an increased level of CD44,^168^ which requires the transformation of CD44v to CD44s isoform.^423^, ^424^ It was reported that 50% of pancreatic cancer tissues were CD44v6‐positive, which indicated a poorer survival in this group of patients.^425^ Currently, two phase I/II clinical trials are ongoing to assess the safety and efficacy of CD44v6 CAR‐T‐cell therapy in patients with breast cancer and other CD44v6‐positive tumors. (NCT04430595 and NCT04427449) Recently, a highly specific CAR against CD44v6 was established aiming to eliminate CD44v6‐expressing HNSCC cells.^426^

## 6 CONCLUSION AND FUTURE PERSPECTIVES

In conclusion, CSCs are a subpopulation of malignant tumor cells with selective capacities for tumor initiation, self‐renewal, metastasis, and unlimited growth into bulks. There is intricate signaling network within CSCs that regulates stemness and biological functions. Thus, targeting pathway molecules that regulate CSCs provides a new option for the treatment of therapy‐resistant or ‐refractory tumors. Meanwhile, extracellular regulating factors, including angiogenic microenvironment, hypoxic microenvironment, TAM, fibroblasts, and a series of protumor paracrine factors, collectively provide a fertile soil that favors CSC growth. Numerous efforts have been undertaken these years to identify such therapies, such as kinase inhibitors and antibodies that block CSC‐associated signaling pathway elements, and some of these approaches have already entered the clinical phase. Furthermore, vaccines, antibodies, and CAR T cells have also expanded the range of CSC‐target therapies.

Obstacles remain regarding the design of CSC‐targeted therapies. Though our understanding of CSCs surface biomarkers has been largely improved in recent years, the surface markers of CSCs may vary according to tumor types and the cell of tumor origin, demonstrating high heterogeneity between tumors or even among cells within one tumor. This heterogeneity has highlighted the challenges in identifying and isolating CSC subpopulations from tumors. Thus, functional assays are recommended to more specifically identify CSCs, including sphere formation capacity in vitro and tumor‐initiation of after transplantation in vivo.

Accumulating evidence now suggests that quiescent CSCs contribute to the refraction of cancers to chemotherapies. Thus, therapeutic approaches that merely inhibit CSC stemness might not be sufficient to suppress postchemotherapy recurrence. The TME plays a critical role in CSC regulation, which not only maintains CSC characteristics via various signals but also facilitates the transition of nonstem cells to stem cell states.^427^ It is thus conceivable that targeting TME components may be more effective in overcoming treatment resistance than directly inhibiting CSCs stemness. However, the heterogeneity of immune cells across cell types has made it difficult to identify the precise CSC‐immune cell interactions. Recently, single‐cell RNA sequencing is extensively used to identify the altering states of CSCs and immune cells, as well as their interactions under different tumor contexts. Moreover, BiAbs that act on both intrinsic regulating factors of CSCs and the CSC‐immune cell crosstalk are recommended.^31^

Finally, in addition to TAMs that have long been identified for their activities in CSC maintenance, recent reports highlight the significance of NK cells in suppressing cancer cell stemness.^290^ CSCs are mostly sensitive to NK cell killing, but in some cases, such as GBM, AML, and breast cancer, CSCs may be resistant to activated NK cells.^428^ However, the anti‐CSC functions of NK cells are suppressed by TAMs, MDSCs (myeloid‐derived suppressor cells), and T‐reg cells.^429^ Future research is required to address the crosstalk between these immune cells in the TME, thereby facilitating the development of more effective CSC‐targeted immunotherapies.

## CONFLICT OF INTERESTS

The authors declared no conflict of interests.

## AUTHOR CONTRIBUTIONS

Wu Min offered the main direction and significant guidance of this manuscript. Wang Manni drafted the manuscript and illustrated the figures for the manuscript. All authors have read and approved the final manuscript.

## ETHICS APPROVAL

Not applicable.

## Data Availability

Not applicable.
